# Voluntary Hospital Staff Conference in Scotland

**Published:** 1921-03-12

**Authors:** Jas. R. Drever

**Affiliations:** Scottish Medical Secretary. 6 Rutland Square, Edinburgh


THE
### EDITOR'S LETTER-BOX.

*Correspondence on all subjects is invited, but we cannot in any way be responsible for the opinions expressed by our correspondents, who must give their name and address as a guarantee of good faith, but not necessarily for publication. Correspondents are reminded that brevity of style and conciseness of statement greatly facilitate early insertion.*

### Voluntary Hospital Staff Conference in Scotland.

*To the Editor of* THE HOSPITAL.

SIR,—Will you allow me to correct an inaccuracy in your report of the above, published in your issue of March 5? The Conference is reported as having passed resolutions advocating contributions by non-necessitous patients, a percentage of these payments to be placed at the disposal of the honorary medical staffs. As a matter of fact, motions to that effect were rejected by the Conference in favour of the following: "That when a hospital receives payment from any public body for the treatment of individual cases, a reasonable proportion of the amount received be put at the disposal of the medical staff."—I am, yours faithfully,

JAS. R. DREVER,

Scottish Medical Secretary.

6 Rutland Square, Edinburgh,
March 7, 1921.

